# The Impact of Ethnicity on Fetal and Maternal Outcomes of Gestational Diabetes

**DOI:** 10.3390/medicina58091161

**Published:** 2022-08-25

**Authors:** Tiziana Filardi, Maria Cristina Gentile, Vittorio Venditti, Antonella Valente, Enrico Bleve, Carmela Santangelo, Susanna Morano

**Affiliations:** 1 Department of Experimental Medicine, “Sapienza” University, Viale del Policlinico 155, 00161 Rome, Italy; 2 Center for Gender-Specific Medicine, Gender Specific Prevention and Health Unit, Istituto Superiore di Sanità, 00161 Rome, Italy

**Keywords:** gestational diabetes mellitus, GDM, race, ethnicity, pregnancy, pregnancy outcomes, pregnancy complications, large for gestational age

## Abstract

*Background and Objectives*: The prevalence of gestational diabetes mellitus (GDM) significantly varies across different ethnic groups. In particular, Africans, Latinos, Asians and Pacific Islanders are the ethnic groups with the highest risk of GDM. The aim of this study was to evaluate the impact of ethnicity on pregnancy outcomes in GDM. *Patients and Methods*: *n* = 399 patients with GDM were enrolled, *n* = 76 patients of high-risk ethnicity (HR-GDM), and *n* = 323 of low-risk ethnicity (LR-GDM). Clinical and biochemical parameters were collected during pregnancy until delivery. Fetal and maternal short-term outcomes were evaluated. *Results:* HR-GDM had significantly higher values of glycosylated hemoglobin checked at 26–29 weeks of gestation (*p* < 0.001). Gestational age at delivery was significantly lower in HR-GDM (*p* = 0.03). The prevalence of impaired fetal growth was significantly higher in HR-GDM than LR-GDM (*p* = 0.009). In logistic regression analysis, the likelihood of impaired fetal growth was seven times higher in HR-GDM than in LR-GDM, after adjustment for pre-pregnancy BMI and gestational weight gain (OR = 7.1 [2.0–25.7] 95% CI, *p* = 0.003). *Conclusions:* HR-GDM had worse pregnancy outcomes compared with LR-GDM. An ethnicity-tailored clinical approach might be effective in reducing adverse outcomes in GDM.

## 1. Introduction

Gestational diabetes mellitus (GDM) refers to “diabetes diagnosed in the second or third trimester of pregnancy that was not clearly overt diabetes prior to gestation” [1]. This condition is broadly diffused worldwide, even though its prevalence is largely influenced by race or ethnicity, as well as by the heterogeneity of the diagnostic criteria adopted in different countries.

It is well known that belonging to specific ethnicities significantly increases the risk of developing GDM. Specifically, high-risk ethnic groups are South and East Asians, Africans, Hispanics, Native Americans and Pacific Islanders [2,3,4,5]. Adverse fetal and maternal outcomes are recognized to be associated with GDM. Perinatal complications encompass impaired fetal growth, which include large for gestational age (LGA, neonatal weight > 90th percentile) and, to a lesser extent, small for gestational age (SGA, neonatal weight < 10th percentile) newborns [6]. Moreover, offspring of women with GDM have increased risk of fetal malformations, rendering antenatal and perinatal management mandatory [7,8]. Maternal short-term complications include preterm delivery, gestational hypertension, preeclampsia and increased rate of caesarean section [9,10]. As for long-term complications, both mothers and infants have an increased likelihood of developing type 2 diabetes (T2D), metabolic syndrome, obesity, and cardiovascular disease (CVD) later in life [9,10,11]. In light of this, prompt diagnosis and appropriate management of GDM, together with long-term follow-up of patients and infants might effectively limit the burden of GDM-related adverse outcomes.

In recent years, the volume of human migration to high income countries has been increasing due to political issues and economic crises. Migration flow includes a large number of women of childbearing age, markedly influencing the patterns of reproductive health in receiving countries [12]. Ethnic minorities carry with them their genetic background and lifestyle habits, which inevitably merge with high income countries’ cultures, increasing the risk of developing cardiovascular and metabolic diseases [13,14]. Although pregnancy and birth are physiological events, migrants might experience substantial language and cultural barriers that limit their access to maternal and neonatal care in destination countries [15]. There is growing concern about the high risk of pregnancy complications and adverse outcomes in ethnic minorities, which urges the adoption of pregnancy monitoring schedules and health policies tailored for ethnic-specific characteristics, in order to reduce the impact of the increased burden of these diseases on healthcare systems.

The understanding of the differences between specific ethnic groups helps in managing pregnancy complications and might considerably limit the burden of GDM. Several retrospective cohort studies have observed ethnic disparities in short-term and long-term outcomes of GDM, reporting conflicting results. In particular, an increased risk of adverse fetal outcomes has been reported in high-risk ethnicities compared to low-risk counterparts [16,17]. However, other studies showed only a modest impact or no effect of race or ethnicity on pregnancy outcomes [18,19,20]. Of note, not all high-risk ethnic groups have been linked to adverse outcomes. Specifically, Asians were consistently reported to have the lowest risk of LGA and macrosomia compared with other ethnicities [21,22,23].

The aim of this study was to evaluate the impact of ethnicity on short-term pregnancy complications, in order to identify relevant risk factors for adverse perinatal outcomes in patients with GDM.

## 2. Patients and Methods

### 2.1. Patients

This study involved *n* = 399 patients with GDM, *n* = 76 of high-risk ethnicity (HR-GDM), and *n* = 323 of low-risk ethnicity (LR-GDM), recruited in the outpatient clinics of Policlinico Umberto I, “Sapienza” University Hospital of Rome, between 2015 and 2021. LR-GDM were all Caucasian, while HR-GDM were 71.1% Asian, 15.8% African, 13.1% Hispanic. All subjects gave their informed consent for inclusion before they participated in the study. The study was conducted in accordance with the Declaration of Helsinki, and the protocol was approved by the Hospital Ethics Committee of “Sapienza” University of Rome (project identification code 3830, date of approval 22 October 2015).

Current recommendations were applied to diagnose GDM [24]. Exclusion criteria were: age < 18 years; pre-pregnancy diabetes (T2D and Type 1 diabetes); alcohol or drug abuse; psychiatric diseases; multiple pregnancy. The patients were evaluated monthly from diagnosis until delivery. A detailed medical history was collected (age, ethnicity, family history, physiological, obstetrical, pharmacological anamnesis, previous diseases). Anthropometric and vital parameters were obtained (weight, BMI, blood pressure, and heart rate). The following laboratory parameters were collected: fasting plasma glucose (FPG), 1-h and 2-h PG after 75-g oral glucose tolerance test (OGTT), glycated haemoglobin (HbA1c), total cholesterol [TC], triglycerides [TG], high density lipoprotein cholesterol [HDL-c], calculated low density lipoprotein cholesterol [LDL-c]), complete blood count. Information about therapy for GDM (diet or insulin) and other therapies (antihypertensive, other drugs) was also collected. Fetal ultrasound parameters at third trimester and delivery outcomes were obtained (gestational week at delivery, type of delivery, neonatal weight, Apgar index, maternal and fetal complications).

A follow-up visit 6–12 weeks after delivery was performed to check post-partum OGTT results.

### 2.2. Statistical Analysis

Mean values ± standard deviations are reported for continuous variables. Frequencies are reported for categorical variables. Kolmogorov–Smirnov testing was used to check for normal distribution of variables. Differences between groups were tested with unpaired sample *t*-testing or Mann–Whitney U testing, according to normal or skewed distribution, respectively. Categorical variables were compared with Fisher’s exact test. Differences between groups were evaluated with a linear model, adjusting for covariates. Univariable and multivariable regression analyses were performed to evaluate the association between the variables of interest, adjusting for confounding factors. A *p*-value < 0.05 was considered statistically significant. Statistical analysis was performed with IBM SPSS Statistics software version 23 (Chicago, IL, USA).

## 3. Results

The clinical and laboratory parameters of the enrolled population are reported in Table 1 and Table 2, respectively. The fetal US parameters at the third trimester are shown in Table 3.

Mean age was significantly lower in HR-GDM than in LR-GDM (31.6 ± 5.0 vs. 34.8 ± 5.4 years, *p* < 0.001)

The prevalence of first-degree family history of T2D was significantly higher in the HR-GDM group than in the LR-GDM group (60.6% vs. 36.1% *p* < 0.001).

A greater percentage of patients in the HR-GDM group had previous GDM, compared with the LR-GDM patients (20.5% vs. 9.0%, *p* = 0.012).

HbA1c values checked at 26–29 weeks of gestation were higher in HR-GDM patients compared with LR-GDM patients (5.6 ± 0.4 vs. 5.3 ± 0.5%, *p* < 0.001).

Delivery data were available for n.28 HR-GDM patients and n.83 LR-GDM patients (Table 4). Gestational age at delivery was significantly lower in HR-GDM than in LR-GDM (37.1 ± 1.3 vs. 38.3 ± 1.8 weeks, *p* = 0.03).

The prevalence of impaired fetal growth was 18% in the whole population (LGA 10.8% and SGA 7.2%), and was significantly higher in HR-GDM (35.7% vs. 12.0%, *p* = 0.009).

According to logistic regression analysis, the likelihood of impaired fetal growth was four times higher in HR-GDM patients than in LR-GDM patients (OR = 4.1 [1.5–11.2] 95% CI, *p* = 0.007). In the multivariate model, HR-GDM was an independent predictor of impaired fetal growth, after adjustment for pre-pregnancy BMI and gestational weight gain (OR = 7.1 [2.0–25.7] 95% CI, *p* = 0.003).

Only 16.1% of patients adhered to the follow-up visit 6–12 weeks after delivery.

## 4. Discussion

GDM can lead to severe pregnancy complications and has become a major public health problem worldwide.

In this study, a sample of pregnant women with GDM were followed-up until delivery and pregnancy outcomes were compared according to ethnicity.

Relevant differences emerged between patients belonging to ethnic groups considered high-risk and low-risk for GDM, both in the baseline risk factors, and in pregnancy outcomes. In particular, the HR-GDM group were significantly younger than LR-GDM patients, despite the comparable prevalence of nulliparity between the two groups. This aspect might mirror relevant cultural differences in approaches to pregnancy across different ethnic groups. Furthermore, a greater proportion of HR-GDM patients had first-degree family history of T2D, which is another major risk factor for GDM. These findings are in line with previous evidence indicating diverse background characteristics between Caucasian and non-Caucasian women with GDM [20,25,26]. Specifically, in a retrospective analysis conducted in Italy, Caucasian GDM patients were significantly older than non-Caucasians [20]. In another study, non-Caucasian women with GDM were younger and had higher prevalence of family history of T2D compared with Caucasian women [26].

It is well established that previous history of GDM markedly increases the incidence of GDM in subsequent pregnancies [27]. In this study, the percentage of women with recurrent GDM was higher in the HR-GDM group compared with the LR-GDM group (20.5% vs. 9%), despite the younger age of HR-GDM patients. Accordingly, although the rate of GDM recurrence is not yet well defined, studies in which the majority of patients belonged to high risk ethnic groups reported higher GDM recurrence rates than studies involving mostly low-risk populations [28].

Although it remained in the normal range, HbA1c measured at GDM diagnosis in the early third trimester was significantly higher in HR-GDM compared with LR-GDM, reflecting higher mean glycemic levels in the previous three months in the high-risk patients. Although OGTT is the gold standard for GDM diagnosis due to its high sensitivity and specificity, it has been observed that HbA1c values in the first trimester of pregnancy are predictive of GDM and postpartum T2D [29,30,31,32]. In addition, several studies have suggested an association between different cut-off levels of HbA1c during pregnancy and adverse outcomes in GDM [29,33,34,35,36]. The mean value of HbA1c decreases by 0.5% in pregnancy compared with pre-pregnancy values, mainly due to the shorter half-life of erythrocytes [37]. In light of this, some studies have tried to define a pregnancy-specific normal range of HbA1c, which seems to stand between 4.3 and 5.4% [38]. Values above this range have previously been associated with poor pregnancy outcomes in GDM [29,33,34,35,36]. Accordingly, compared with LR-GDM women in this study, HR-GDM had mean values of HbA1c above this range and a more frequent occurrence of birth weight impairment. In light of this, further studies are necessary to define the role of HbA1c in predicting GDM and adverse outcomes. In particular, a pregnancy-specific HbA1c cut-off might have clinical usefulness for identifying at an early stage pregnancies with increased risk of GDM complications, helping physicians in tailoring GDM management according to risk stratification.

In this study, impaired fetal growth was more frequent in HR-GDM patients than in LR-GDM patients, even after adjustment for confounding factors. In recent decades, a growing number of studies have reported ethnic disparities in the development of GDM complications. Most of these studies showed that high-risk ethnicities were associated with a greater risk of poor pregnancy outcomes, compared with low-risk ethnicities. In particular, Silva et al. observed that Pacific Islanders and Filipinos had increased prevalence of macrosomia compared with Japanese, Chinese, and Caucasian women [17]. Similarly, in another retrospective study, non-Caucasian patients had more frequent occurrence of macrosomia and LGA, regardless of confounding factors such as BMI and maternal glycemic control [39]. Of note, the risk of adverse perinatal outcomes seems to vary substantially even across high-risk ethnicities. In particular, Asian women with GDM, especially South Asians, have been consistently reported to have lower risk of LGA and macrosomia compared with other ethnic groups [21,23,30,40]. Some studies have found that non-Hispanic black women with GDM had the highest risk of LGA [21,23,30,40]. Meanwhile, in another study, the highest risk of Caesarean section and perinatal complications (LGA, neonatal hypoglycemia) was associated with Hispanic ethnicity compared with other groups, including African patients [16]. However, the proportion of black patients included in the latter analysis was relatively low. On the other hand, not all studies have observed relevant differences in perinatal outcomes among different ethnic groups [19,20].

Several studies have focused on other outcomes such as incident diabetes after GDM, reporting conflicting results. Specifically, Shen et al. observed that Chinese women with previous GDM had the greatest risk of T2D in the following 10 years, whereas African American and Caucasian women had intermediate and low risk, respectively [41]. Conversely, in a recent meta-analysis including more than 80,000 women with previous GDM, the highest incidence of T2D after GDM was associated with black ethnicity [42,43]. In our study, only a small proportion of women underwent post-partum screening (16.1%). Due to the lack of data about OGTT 6–12 weeks after delivery, the incidence of post-partum diabetes in this population could not be estimated. Overall, rates of recommended post-partum screening are reportedly low (around 40–50%) [42,43,44] and seem to be associated with several socio-cultural factors, including education, parity, and race or ethnicity [44]. These results suggest the need for urgent strategies to increase post-partum follow-up adherence, in order to reduce the burden of long-term maternal and fetal complications of GDM, namely T2D and cardiovascular diseases, through appropriate preventive intervention programs.

Overall, in light of these findings, specific ethnic groups that are at high risk of complications might benefit from more tailored education and intervention strategies and follow-up programs in the management of GDM. Moreover, the monitoring of HbA1c might be of clinical utility to avoid poor maternal and fetal outcomes in pregnant women with GDM.

The main limitations of this study were the retrospective nature of the analysis and the small number of patients included in the evaluation at term and after delivery, which calls for cautious interpretation. Future studies with larger sample sizes and longer follow-ups are mandatory to confirm these findings.

## 5. Conclusions

Ethnic groups at high risk of developing GDM had worse glycemic control at GDM diagnosis, lower gestational age at delivery, and increased risk of impaired fetal growth.

Given the implications of poor perinatal outcomes associated with GDM, the adoption of ethnic-specific management strategies might be useful in clinical practice.

## Figures and Tables

**Table 1:** Clinical parameters.

	HR-GDM *n* = 76	LR-GDM *n* = 399	*p*-Value
Age (years)	31.6 ± 5.0	34.8 ± 5.4	<0.001 *
Gestational week at enrolment (n)	27.4 ± 6.1	27.6 ± 5.3	0.78
Pre-pregnancy BMI (kg/m^2^)	26.6 ± 6.0	26.6 ± 6.7	0.97
3rd trimester BMI (kg/m^2^)	29.9 ± 6.6	30.0 ± 6.3	0.92
Family history of T2D (%)	60.6	36.1	<0.001 *
Previous GDM (%)	20.5	9.0	0.012 *
Nulliparity (%)	26.0	34.3	0.21
Previous miscarriages (%)	24.7	37.6	0.17 ‡
Insulin therapy (%)	50.0	40.1	0.15
Gestational hypertension (%)	5.4	8.1	0.63

HR-GDM: High-risk ethnicity; LR-GDM: low-risk ethnicity; BMI: body mass index; T2D: type 2 diabetes; SBP: systolic blood pressure; DBP: diastolic blood pressure; * *p* < 0.05; ‡ Adjusted for age. Data are expressed as mean ± standard deviation or as frequencies.

**Table 2:** Laboratory parameters.

	HR-GDM *n* = 76	LR-GDM *n* = 399	*p*-Value
FPG (mg/dL)	99.6 ± 16.3	92.5 ± 14.7	0.18
Gestational week OGTT (n)	23.1 ± 5.6	24.6 ± 4.7	0.035 *
Glycaemia T0 16–18 w (mg/dL)	94.9 ± 14.8	92.9 ± 11.1	0.55
Glycaemia T60 16–18 w (mg/dL)	188.1 ± 31.3	171.7 ± 40.6	0.14
Glycaemia T120 16–18 w (mg/dL)	146.6 ± 34.9	144.8 ± 36.7	0.85
Glycaemia T0 24–28 w (mg/dL)	91.3 ± 11.8	88.0 ± 11.5	0.09
Glycaemia T60 24–28 w (mg/dL)	177.6 ± 35.0	176.9 ± 32.5	0.90
Glycaemia T120 24–28 w (mg/dL)	153.9 ± 30.7	149.4 ± 34.5	0.42
HbA1c (26–29 w) (%)	5.6 ± 0.4	5.3 ± 0.5	<0.001 *
TC (mg/dL)	230.2 ± 46.9	252.5 ± 57.5	0.06
LDL-c (mg/dL)	120.4 ± 43.6	140.3 ± 49.4	0.056
HDL-c (mg/dL)	63.2 ± 16.7	72.0 ± 26.4	0.13
Triglycerides (mg/dL)	233.5 ± 60.1	222.5 ± 101.8	0.59

HR-GDM: High-risk ethnicity; LR-GDM: low-risk ethnicity; w: weeks; FPG: fasting plasma glucose; OGTT: oral glucose tolerance test; HbA1c: glycated hemoglobin; TC: total cholesterol; LDL-c: LDL-cholesterol; HDL-c: HDL-cholesterol; * *p* < 0.05; Data are expressed as mean ± standard deviation.

**Table 3:** Fetal ultrasound parameters (third trimester).

	HR-GDM *n* = 76	LR-GDM *n* = 399	*p*-Value
Gestational week (n)	29.2 ± 4.9	30.1 ± 4.3	0.34
BPD (mm)	72.6 ± 13.6	76.6 ± 14.0	0.24
HC (mm)	255.3 ± 52.0	280.8 ± 38.8	0.06
AC (mm)	247.1 ± 66.1	264.6 ± 61.0	0.24
FL (mm)	55.4 ± 12.5	58.4 ± 9.8	0.24
HL (mm)	49.1 ± 11.7	52.5 ± 7.5	0.16
EFW (g)	2033.2 ± 467.0	1911.8 ± 564.0	0.48

HR-GDM: High-risk ethnicity; LR-GDM: low-risk ethnicity; BPD: biparietal diameter; HC: head circumference; AC: abdominal circumference; FL: femur length; HL: humeral length; EFW: estimated fetal weight; data are expressed as mean ± standard deviation.

**Table 4:** Neonatal parameters and pregnancy outcomes.

	HR-GDM *n* = 28	LR-GDM *n* = 83	*p*-Value
Gestational week delivery (n)	37.1 ± 1.3	38.3 ± 1.8	0.009 *
Caesarean section (%)	72.2	66.7	0.78
Impaired growth	35.7	12.0	0.009 *
LGA (%)	21.4	7.2	-
SGA (%)	14.3	4.8	-
Apgar score	8.6 ± 0.6	9.0 ± 0.9	0.07
Hypoglycemia (%)	16.7	2.4	0.13
Jaundice (%)	8.3	2.5	0.41
ARDS (%)	0	2.4	1.00

HR-GDM: High-risk ethnicity; LR-GDM: low-risk ethnicity; LGA: large for gestational age; SGA: small for gestational age; ARDS: acute respiratory distress syndrome; * *p* < 0.05; Data are expressed as mean ± standard deviation or as frequencies.

## Data Availability

The data presented in this study are available on request from the corresponding author.

## References

[1] Draznin B., Aroda V.R., Bakris G., Benson G., Brown F.M., Freeman R., Green J., Huang E., American Diabetes Association Professional Practice Committee (2022). 2. Classification and Diagnosis of Diabetes: Standards of Medical Care in Diabetes-2022. Diabetes Care.

[2] Berkowitz G.S., Lapinski R.H., Wein R., Lee D. (1992). Race/ethnicity and other risk factors for gestational diabetes. Am. J. Epidemiol..

[3] Dornhorst A., Paterson C.M., Nicholls J.S., Wadsworth J., Chiu D.C., Elkeles R.S., Johnston D.G., Beard R.W. (1992). High prevalence of gestational diabetes in women from ethnic minority groups. Diabet. Med..

[4] Metzger B.E., Coustan D.R. (1998). Summary and recommendations of the Fourth International Workshop-Conference on Gestational Diabetes Mellitus. The Organizing Committee. Diabetes Care.

[5] Yuen L., Wong V.W., Simmons D. (2018). Ethnic Disparities in Gestational Diabetes. Curr. Diab. Rep..

[6] Metzger B.E., Lowe L.P., Dyer A.R., Trimble E.R., Chaovarindr U., Coustan D.R., Hadden D.R., McCance D.R., Hod M., Hapo Study Cooperative Research Group (2008). Hyperglycemia and adverse pregnancy outcomes. N. Engl. J. Med..

[7] Zhang T.N., Huang X.M., Zhao X.Y., Wang W., Wen R., Gao S.Y. (2022). Risks of specific congenital anomalies in offspring of women with diabetes: A systematic review and meta-analysis of population-based studies including over 80 million births. PLoS Med..

[8] Quaresima P., Homfray T., Greco E. (2019). Obstetric complications in pregnancies with life-limiting malformations. Curr. Opin. Obstet. Gynecol..

[9] Pintaudi B., Fresa R., Dalfra M., Dodesini A.R., Vitacolonna E., Tumminia A., Sciacca L., Lencioni C., Marcone T., Lucisano G. (2018). The risk stratification of adverse neonatal outcomes in women with gestational diabetes (STRONG) study. Acta Diabetol..

[10] Xiang A.H., Li B.H., Black M.H., Sacks D.A., Buchanan T.A., Jacobsen S.J., Lawrence J.M. (2011). Racial and ethnic disparities in diabetes risk after gestational diabetes mellitus. Diabetologia.

[11] Kelstrup L., Damm P., Mathiesen E.R., Hansen T., Vaag A.A., Pedersen O., Clausen T.D. (2013). Insulin resistance and impaired pancreatic beta-cell function in adult offspring of women with diabetes in pregnancy. J. Clin. Endocrinol. Metab..

[12] Balaam M.C., Haith-Cooper M., Parizkova A., Weckend M.J., Fleming V., Roosalu T., Vrzina S.S. (2017). A concept analysis of the term migrant women in the context of pregnancy. Int. J. Nurs. Pract..

[13] Tillin T., Forouhi N., Johnston D.G., McKeigue P.M., Chaturvedi N., Godsland I.F. (2005). Metabolic syndrome and coronary heart disease in South Asians, African-Caribbeans and white Europeans: A UK population-based cross-sectional study. Diabetologia.

[14] Satia-Abouta J., Patterson R.E., Neuhouser M.L., Elder J. (2002). Dietary acculturation: Applications to nutrition research and dietetics. J. Am. Diet. Assoc..

[15] Song J.E., Ahn J.A., Kim T., Roh E.H. (2016). A qualitative review of immigrant women’s experiences of maternal adaptation in South Korea. Midwifery.

[16] Hernandez-Rivas E., Flores-Le Roux J.A., Benaiges D., Sagarra E., Chillaron J.J., Paya A., Puig-de Dou J., Goday A., Lopez-Vilchez M.A., Pedro-Botet J. (2013). Gestational diabetes in a multiethnic population of Spain: Clinical characteristics and perinatal outcomes. Diabetes Res. Clin. Pract..

[17] Silva J.K., Kaholokula J.K., Ratner R., Mau M. (2006). Ethnic differences in perinatal outcome of gestational diabetes mellitus. Diabetes Care.

[18] Seghieri G., Di Cianni G., Seghieri M., Lacaria E., Corsi E., Lencioni C., Gualdani E., Voller F., Francesconi P. (2020). Risk and adverse outcomes of gestational diabetes in migrants: A population cohort study. Diabetes Res. Clin. Pract..

[19] Mocarski M., Savitz D.A. (2012). Ethnic differences in the association between gestational diabetes and pregnancy outcome. Matern. Child Health J..

[20] Caputo M., Bullara V., Mele C., Sama M.T., Zavattaro M., Ferrero A., Daffara T., Leone I., Giachetti G., Antoniotti V. (2021). Gestational Diabetes Mellitus: Clinical Characteristics and Perinatal Outcomes in a Multiethnic Population of North Italy. Int. J. Endocrinol..

[21] Wan C.S., Abell S., Aroni R., Nankervis A., Boyle J., Teede H. (2019). Ethnic differences in prevalence, risk factors, and perinatal outcomes of gestational diabetes mellitus: A comparison between immigrant ethnic Chinese women and Australian-born Caucasian women in Australia. J. Diabetes.

[22] Kwong W., Ray J.G., Wu W., Feig D.S., Lowe J., Lipscombe L.L. (2019). Perinatal Outcomes Among Different Asian Groups With Gestational Diabetes Mellitus in Ontario: A Cohort Study. Can. J. Diabetes.

[23] Esakoff T.F., Caughey A.B., Block-Kurbisch I., Inturrisi M., Cheng Y.W. (2011). Perinatal outcomes in patients with gestational diabetes mellitus by race/ethnicity. J. Matern. Fetal Neonatal Med..

[24] Associazione Medici Diabetologi—Società Italiana di Diabetologia Standard Italiani per La Cura Del Diabete Mellito 2018. http://www.siditalia.it/clinica/standard-di-cura-amd-sid.

[25] Bordin P., Dotto L., Battistella L., Rosso E., Pecci L., Valent F., Collarile P., Vanin M. (2020). Gestational diabetes mellitus yesterday, today and tomorrow: A 13 year italian cohort study. Diabetes Res. Clin. Pract..

[26] Read S.H., Wu W., Ray J.G., Lowe J., Feig D.S., Lipscombe L.L. (2019). Characteristics of Women with Gestational Diabetes From Non-Caucasian Compared With Caucasian Ethnic Groups. Can. J. Diabetes.

[27] Morikawa M., Yamada T., Saito Y., Noshiro K., Mayama M., Nakagawa-Akabane K., Umazume T., Chiba K., Kawaguchi S., Watari H. (2021). Predictors of recurrent gestational diabetes mellitus: A Japanese multicenter cohort study and literature review. J. Obstet. Gynaecol. Res..

[28] Egan A.M., Enninga E.A.L., Alrahmani L., Weaver A.L., Sarras M.P., Ruano R. (2021). Recurrent Gestational Diabetes Mellitus: A Narrative Review and Single-Center Experience. J. Clin. Med..

[29] Kattini R., Hummelen R., Kelly L. (2020). Early Gestational Diabetes Mellitus Screening with Glycated Hemoglobin: A Systematic Review. J. Obstet. Gynaecol. Can..

[30] Kwon S.S., Kwon J.Y., Park Y.W., Kim Y.H., Lim J.B. (2015). HbA1c for diagnosis and prognosis of gestational diabetes mellitus. Diabetes Res. Clin. Pract..

[31] Renz P.B., Chume F.C., Timm J.R.T., Pimentel A.L., Camargo J.L. (2019). Diagnostic accuracy of glycated hemoglobin for gestational diabetes mellitus: A systematic review and meta-analysis. Clin. Chem. Lab. Med..

[32] Valadan M., Bahramnezhad Z., Golshahi F., Feizabad E. (2022). The role of first-trimester HbA1c in the early detection of gestational diabetes. BMC Pregnancy Childbirth.

[33] Arbib N., Shmueli A., Salman L., Krispin E., Toledano Y., Hadar E. (2019). First trimester glycosylated hemoglobin as a predictor of gestational diabetes mellitus. Int. J. Gynaecol. Obstet..

[34] Fong A., Serra A.E., Gabby L., Wing D.A., Berkowitz K.M. (2014). Use of hemoglobin A1c as an early predictor of gestational diabetes mellitus. Am. J. Obstet. Gynecol..

[35] Hughes R.C., Moore M.P., Gullam J.E., Mohamed K., Rowan J. (2014). An early pregnancy HbA1c >/=5.9% (41 mmol/mol) is optimal for detecting diabetes and identifies women at increased risk of adverse pregnancy outcomes. Diabetes Care.

[36] Mane L., Flores-Le Roux J.A., Pedro-Botet J., Gortazar L., Chillaron J.J., Llaurado G., Paya A., Benaiges D. (2019). Is fasting plasma glucose in early pregnancy a better predictor of adverse obstetric outcomes than glycated haemoglobin?. Eur. J. Obstet. Gynecol. Reprod. Biol..

[37] Radder J.K., van Roosmalen J. (2005). HbA1c in healthy, pregnant women. Neth. J. Med..

[38] O’Connor C., O’Shea P.M., Owens L.A., Carmody L., Avalos G., Nestor L., Lydon K., Dunne F. (2011). Trimester-specific reference intervals for haemoglobin A1c (HbA1c) in pregnancy. Clin. Chem. Lab. Med..

[39] Aulinas A., Biagetti B., Vinagre I., Capel I., Ubeda J., Maria M.A., Garcia-Patterson A., Adelantado J.M., Ginovart G., Corcoy R. (2013). Gestational diabetes mellitus and maternal ethnicity: High prevalence of fetal macrosomia in non-Caucasian women. Med. Clin..

[40] Xiang A.H., Black M.H., Li B.H., Martinez M.P., Sacks D.A., Lawrence J.M., Buchanan T.A., Jacobsen S.J. (2015). Racial and ethnic disparities in extremes of fetal growth after gestational diabetes mellitus. Diabetologia.

[41] Shen Y., Hou L., Liu H., Wang L., Leng J., Li W., Hu G. (2019). Racial differences of incident diabetes postpartum in women with a history of gestational diabetes. J. Diabetes Complicat..

[42] Herrick C.J., Puri R., Rahaman R., Hardi A., Stewart K., Colditz G.A. (2020). Maternal Race/Ethnicity and Postpartum Diabetes Screening: A Systematic Review and Meta-Analysis. J. Womens Health.

[43] Wang Y., Chen L., Horswell R., Xiao K., Besse J., Johnson J., Ryan D.H., Hu G. (2012). Racial differences in the association between gestational diabetes mellitus and risk of type 2 diabetes. J. Womens Health.

[44] Brown S.D., Hedderson M.M., Zhu Y., Tsai A.L., Feng J., Quesenberry C.P., Ferrara A. (2022). Uptake of guideline-recommended postpartum diabetes screening among diverse women with gestational diabetes: Associations with patient factors in an integrated health system in USA. BMJ Open Diabetes Res. Care.

